# Direct Spectroscopy for Probing the Critical Role of Partial Covalency in Oxygen Reduction Reaction for Cobalt-Manganese Spinel Oxides

**DOI:** 10.3390/nano9040577

**Published:** 2019-04-09

**Authors:** Xinghui Long, Pengfei Yu, Nian Zhang, Chun Li, Xuefei Feng, Guoxi Ren, Shun Zheng, Jiamin Fu, Fangyi Cheng, Xiaosong Liu

**Affiliations:** 1 State Key Laboratory of Functional Materials for Informatics, Shanghai Institute of Microsystem and Information Technology, Chinese Academy of Sciences, Shanghai 200050, China; xhlong@mail.sim.ac.cn (X.L.); ypfaq@mail.sim.ac.cn (P.Y.); zhangn@mail.sim.ac.cn (N.Z.); gxren@mail.sim.ac.cn (G.R.); shunzheng@mail.sim.ac.cn (S.Z.); fujm@shanghaitech.edu.cn (J.F.); 2 CAS Center for Excellence in Superconducting Electronics (CENSE), Chinese Academy of Sciences, Shanghai 200050, China; 3 University of Chinese Academy of Sciences, Beijing 100049, China; 4 Key Laboratory of Advanced Energy Materials Chemistry (Ministry of Education) and State Key Laboratory of Elemento-Organic Chemistry, College of Chemistry, Nankai University, Tianjin 300071, China; kemistlic@foxmail.com (C.L.); fycheng@nankai.edu.cn (F.C.); 5 Advanced Light Source, Lawrence Berkeley National Laboratory, Berkeley, CA 94720, USA; xuefeifeng2013@gmail.com; 6 School of Physical Science and Technology, Shanghai Tech University, Shanghai 200031, China

**Keywords:** oxygen reduction reaction, spinel oxides, soft X-ray absorption spectroscopy, partial covalency, catalytic activity

## Abstract

Nanocrystalline multivalent metal spinels are considered as attractive non-precious oxygen electrocatalysts. Identifying their active sites and understanding their reaction mechanisms are essential to explore novel transition metal (TM) oxides catalysts and further promote their catalytic efficiency. Here we report a systematic investigation, by means of soft X-ray absorption spectroscopy (sXAS), on cubic and tetragonal Co_x_Mn_3-x_O_4_ (x = 1, 1.5, 2) spinel oxides as a family of highly active catalysts for the oxygen reduction reaction (ORR). We demonstrate that the ORR activity for oxide catalysts primarily correlates to the partial covalency of between O 2p orbital with Mn^4+^ 3d t_2g_-down/e_g_-up, Mn^3+^ 3d e_g_-up and Co^3+^ 3d e_g_-up orbitals in octahedron, which is directly revealed by the O K-edge sXAS. Our findings propose the critical influences of the partial covalency between oxygen 2p band and specific metal 3d band on the competition between intermediates displacement of the ORR, and thus highlight the importance of electronic structure in controlling oxide catalytic activity.

## 1. Introduction

The oxygen reduction reaction (ORR) and/or oxygen evolution reaction (OER) on an oxygen-based electrode are essential for a wide range of electrochemical energy conversion and storage technologies, such as direct solar cell [1], electrolytic water splitting [2], rechargeable metal–air batteries [3], and regenerative fuel cells [4]. However, the intrinsic slow kinetics of ORR/OER is the obstacle for their application. It is a great challenge to seek for highly active catalysts to improve the efficiency of ORR and OER. To date, the best known catalysts for oxygen electrocatalysis are Pt-alloy catalysts for the ORR [5] and iridium-oxide- or ruthenium-oxide-based catalysts for the OER [6]. Unfortunately, the scarce crustal abundance of the noble metals limits their commercial viability. Transition metal (TM) oxides and carbon materials with excellent electrocatalysts and high stability [7,8,9], owing many advantages such as high abundance, low-cost, easy prepared, and environmental friendliness, are considered as an alternative to noble metals. In particularly, spinel oxides have been widely used as catalyst for ORR and/or OER [10,11,12,13,14,15,16]. In pursuit of further enhanced oxygen electrocatalytic activity, it is necessary to understand the catalytic mechanism of TM oxides and to identify the activity site, which has attracted extensive research efforts.

For instance, Rios et al. found that the surface Co^3+^ in Mn_x_Co_3-x_O_4_ (x > 0) was the active site, which made Co_3_O_4_ the most active for OER [17]. On the contrary, Restovic et al. pointed out that the electrocatalytic activity in Mn_x_Co_3-x_O_4_ of the ORR was correlated to the Mn content, and more precisely to the amount of Mn^4+^/Mn^3+^ pairs [18]. These results suggested that two metals in dual-metal spinel system and their redox pairs play different roles in influencing the catalytic performances for ORR or OER. Based on a systematic study of 3d TM perovskite oxides, Shao-Horn Yang et al. discovered a volcano shape relationship between the e_g_-filling descriptor, depicting filling degree of the surface active ions e_g_ orbital, and the catalytic activity of ORR/OER [19,20]. Almost at the same time, Zhichuan J. Xu et al. speculated that the e_g_ occupancy of the active cation in the octahedral site is the activity descriptor for the ORR/OER of spinels [21,22]. They also elucidated, based on an investigation of the composition dependence of ORR in ZnCo_x_Mn_2−x_O_4_ (x = 0.0–2.0) spinel, that the modulated e_g_ occupancy of active Mn cations, as a consequence of the superexchange effect between edge sharing [CoO_6_] and [MnO_6_] octahedra, correlated to the ORR activity [22]. However, David N. Mueller et al. revealed that the oxygen anions near the surface rather than the TM cations were a significant redox partner to molecular oxygen because of the strong covalency between oxygen 2p orbital and TM 3d orbital in oxygen-deficient perovskite oxides [23]. In addition, the covalency between metal-d and oxygen-p had been reported to play a critical role in increasing the activities of oxygen electrocatalysis [19,24]. Obviously, although the spinel oxides have been extensively studied, it is still under debate about their catalytic mechanism of ORR or OER activity, especially for dual-metal spinel oxides.

To tackle this long-standing and important fundamental problem, we herein report a systematic and detailed study of a series of nanocrystalline dual-metal spinals Co_x_Mn_3-x_O_4_ through a detailed study with soft X-ray absorption spectroscopy (sXAS). The aforementioned facile synthesis methodology [25] facilitates selective formation of cubic or tetragonal phases and various compositions of Co_x_Mn_3-x_O_4_ (x = 1, 1.5, 2) nanoparticles, which are believed as two main factors affecting their ORR catalytic activities. The subtle variation in TM L-edge spectra for both cubic and tetrahedral samples indicates very little valance change of TM with the variation of the component proportion. Surprisingly, the notable differences are observed, associated with the substitution of Co by Mn, from O K-edge absorption spectra, in particular the pre-edge structures that arise from the covalent mixture of metal-3d and oxygen-2p electronic states. These covalent characteristics is analyzed in depth through the deconvolution of pre-edge features and comparison to a series of reference samples to identify their origins. Our results suggest that the partial covalency of O 2p orbital with Mn^4+^ 3d t_2g_-down/e_g_-up, Mn^3+^ 3d e_g_-up and Co^3+^ 3d e_g_-up orbitals have stronger correlation than other orbitals to the ORR catalytic activities. These findings may provide a new experimental evidence from the point of view of electronic structure to unveil the catalytic mechanism of dual-metal spinels.

## 2. Experimental Method

### 2.1. Synthesis of Co_x_Mn_3-x_O_4_ Oxides and Electrochemical Characterization

The Co_x_Mn_3-x_O_4_ oxides were obtained by a solution synthesis method. In a typical synthesis of Co_x_Mn_3-x_O_4_ spinel oxide, Co(NO_3_)_2_ and Mn(NO_3_)_2_ (Sigma Aldrich, St. Louis, MO, USA) were used as precursor of cobalt and manganese, respectively. The specific steps can be summarized as stirring the solution containing aqueous ammonia (Sigma Aldrich, St. Louis, MO, USA), Co(NO_3_)_2_ and Mn(NO_3_)_2_ solution, then evaporating by heating to obtain the final spinel oxide. Different ratios x and phase structure were obtained by controlling the molar ratio of cobalt and manganese precursor and the order of adding ammonia water, Co(NO_3_)_2_ and Mn(NO_3_)_2_ solution, respectively.

To test the electrochemical performance, a three electrodes electrochemical cell were used, which contains a calomel reference electrode, a Pt counter electrode and a working electrode, respectively. Catalyst ink, containing spinels oxide, carbon powder, water, isopropyl alcohol and neutralized Nafion solution (Sigma Aldrich, St. Louis, MO, USA), was pipetted on the glassy carbon electrode to form the working electrode. The PARSTAT 263A workstation (AMETEK, Berwyn, PA, USA) accompanied with a model 636 system (AMETEK, Berwyn, PA, USA) was used to record the voltammetry data with a potential scan rate of 5 mV s^−1^. Measurements were carried out in 0.1 M aqueous KOH saturated with either purified Ar or O_2_ at room temperature. All potentials were calibrated with reference to standard reversible hydrogen electrode. The detailed experimental methods can refer to reference [26].

### 2.2. Soft X-ray Absorption Spectroscopy (sXAS)

sXAS were performed at beamline 20A1 of National Synchrotron Radiation Research Center (NSRRC) in Hsinchu, Taiwan. The storage ring was operated with energy of 1.5 GeV and a current of 300 mA. The beamline was equipped with a 6-m high-energy spherical grating monochromator (6m-HSGM) to supply a photon beam with resolving power up to 8000 [27]. The spectra were collected in total electron yield (TEY) mode in an under ultrahigh-vacuum (UHV) chamber with a base pressure about 5 × 10^−10^ Torr, corresponding to probe depth of about 10 nm. All the spectra have been normalized to the photocurrent from the upstream clean gold mesh to eliminate the fluctuation of the beam flux. The photon energy was calibrated with the spectra of reference samples (MnO for Mn L-edge, CoO for Co L-edge and SrTiO_3_ for O K-edge) measured simultaneously.

## 3. Results and Discussion

**Figure 1:**
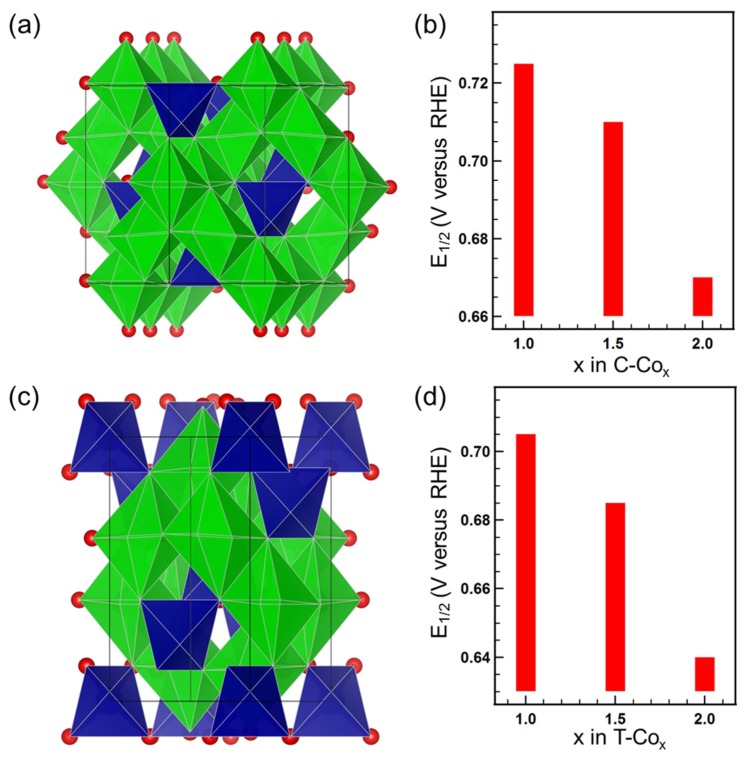
(**a**,**c**) Structure diagram of cubic and tetragonal spinel oxides, respectively. The red ball, blue polyhedron, and green polyhedron represent the oxygen anion, tetrahedron, and octahedron, respectively. (**b**,**d**) The half-wave potential E_1/2_ versus the Co content x in the cubic and tetragonal series, respectively. (5 mV s^−1^ scan rate).

Cubic and tetragonal spinel Co_x_Mn_3-x_O_4_ (x = 1, 1.5, 2) samples (labeled as C–Co_1_, C–Co_1.5_, C–Co_2_, T–Co_1_, T–Co_1.5_, and T–Co_2_, respectively) are obtained by solution synthesis method and their crystal structure are shown in Figure 1a,c. Spinel oxides have A[B_2_]X_4_ molecular formula, in which A stands for cations occupied eighth of the tetrahedral sites, B stands for cations occupied half of the octahedral sites, and X represents oxygen anions with a close-packed structure. The structure, morphology and ORR performance of Co_x_Mn_3-x_O_4_ (x = 1, 1.5, 2) can be found in the previous report [26]. The different ORR performance can be viewed from the half-wave potential E_1/2_ versus reversible hydrogen electrode (RHE) acquired from the polarization profiles, as shown in Figure 1b,d. For both cubic and tetrahedral phases, the activities decrease with the increase of Co/Mn ratio, i.e., CoMn_2_ > Co_1.5_Mn_1.5_ > Co_2_Mn. The results indicate that lower Co/Mn ratio are more favorable to intrinsic catalytic activity.

**Figure 2:**
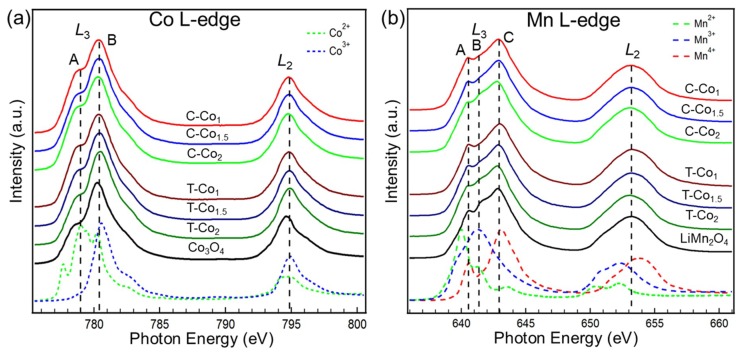
(**a**) Co L-edge soft X-ray absorption spectroscopy (sXAS) spectra of the spinel oxides with Co^2+^ (CoO), Co^3+^ (LiCoO_2_) and Co_3_O_4_. (**b**) Mn L-edge sXAS spectra of spinel oxides with Mn^2+^ (MnO), Mn^3+^ (Mn_2_O_3_), Mn^4+^ (Li_2_MnO_3_), and LiMn_2_O_4_.

To reveal the effect of the Co/Mn ratio on the electronic structure and get a deep insight into its relationship with the catalytic performance, TM L-edge sXAS spectra are firstly studied. The sXAS spectra of Co and Mn L-edge of cubic and tetragonal Co_x_Mn_3-x_O_4_ (x = 1, 1.5, 2) under UHV condition are shown in Figure 2a,b, respectively. It can be seen that both the Co and Mn L-edge spectra split into two separate sets of peaks named as L_3_ and L_2_-edge as a result of the 2p spin-orbital coupling interaction. We herein focus on the evolution of L_3_-edge because of the stronger intensities and refined features compared to L_2_-edge. Based on the reference samples with different valence states and published results [28,29,30,31], the peak A (~779 eV) and peak B (~780.6 eV) are assigned to the Co^2+^ and Co^3+^ states, respectively. The overall lineshape indicates that the Co of Co_x_Mn_3-x_O_4_ primarily exist in a mixed 2+/3+ oxidation state. The subtle spectral variations with different Co/Mn ratios imply little valance changes of Co for both the cubic and tetragonal phases. Moreover, all spectra of Co_x_Mn_3-x_O_4_ are very similar to the spectrum of spinel Co_3_O_4_, suggesting that the Co^2+^ and Co^3+^ occupy the tetrahedral and octahedral sites, respectively [28,32,33,34]. The Mn L-edge sXAS spectra show similar phenomena. Following the same analysis, we are able to identify that the oxidations of Mn in Co_x_Mn_3-x_O_4_ primarily exist in a mixed 3+/4+ state in comparison with previous studies [29,35,36,37]. The similarity of spectra between Co_x_Mn_3-x_O_4_ and spinel LiMn_2_O_4_ reveals that both Mn^3+^ and Mn^4+^ occupy the octahedral sites. More distinct variation with the alteration of x in Co_x_Mn_3-x_O_4_ are observed in the Mn L-edge spectra than the Co L-edge. As the valance state of Mn is higher than Co, the valance changes state that TM or O vacancies are created during the synthetic process, which is consistent with previous report [26]. Besides, it has been well established that the high spin states correspond to large branching ratio I(L_3_)/[I(L_3_) + I(L_2_)] [38,39]. The small branching ratio at Co L-edge and large in Mn L-edge clear that Co stays at low-spin state [31] and Mn at high-spin state [40] in all samples, respectively.

**Figure 3:**
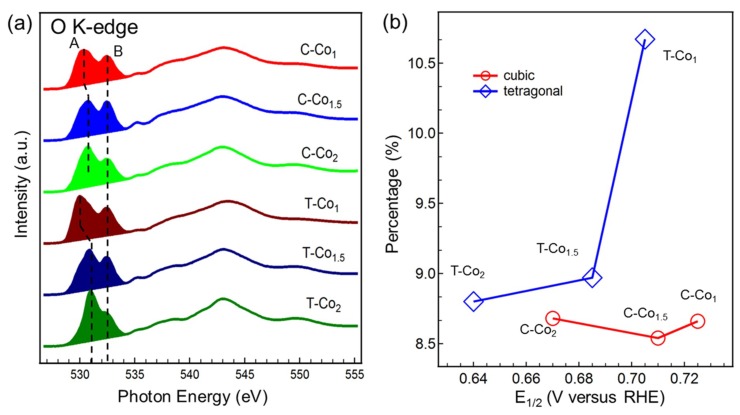
The role of M 3d-O 2p covalency on the oxygen reduction reaction (ORR) activity of Co_x_Mn_3-x_O_4_ spinel oxides. (**a**) O K-edge sXAS spectra of cubic and tetragonal Co_x_Mn_3-x_O_4_ (x = 1, 1.5, 2). Reproduced with permission from [26]. Copyright Nature Publishing Group, 2015. (**b**) the normalized absorbance percentage (absorbance percentage from the shaded section in A) versus the half-wave potential E_1/2_.

Since very little changes from TM L-edge are observed with the alteration of Co/Mn ratio, O K-edge is examined to further track the possible evolution of electronic structure. The O K-edge sXAS spectra of the spinel oxides in Figure 3a can be divided into two regions. The first region (529 ~ 535 eV) shown in the shade, so-called pre-edge, is primarily associated with the O 1s to unoccupied O 2p-TM 3d hybridized states. The second region (above 535 eV) has been attributed to the excitations of O 1s to O 2p−TM 4sp states [23,24,29,41]. Different from the TM L-edge, a significant change is observed in the O 2p−TM 3d region of O K-edge. The energy position of the peak A shifts to high energy as the cobalt content x increases, and the intensity ratio between peak A and B also changes for both cubic and tetragonal phases. This suggests that the M 3d-O 2p covalency may be regulated by changing the ratio x in Co_x_Mn_3-x_O_4_ oxides. In order to further testify if there exists an ORR activity-determining factor related to M 3d-O 2p covalency, we investigate how the ORR activity and M 3d-O 2p covalency changes as a function of x in Co_x_Mn_3-x_O_4_. The normalized absorbance percentage, which is estimated by the percentage of shaded area with subtracting a linear background relative to the entire area of the curve showed in Figure 3a, is used to quantify the strength of M 3d-O 2p covalency. To identify the effect of the covalency of M 3d-O 2p on the ORR activity, we plot the normalized absorbance percentage versus the half-wave potential E_1/2_ in Figure 3b. The M 3d-O 2p covalency exhibits a consistent variation trend with the ORR performance in the tetragonal phase, which demonstrates that increasing the M 3d-O 2p covalency positively affects ORR activity. On the contrary, this correlation cannot be observed in the cubic phase. It evidenced that the strength of the M 3d-O 2p covalency is not a common descriptor of the ORR performance for both the cubic and tetragonal phases. More complicated underlying mechanism may play more crucial role to determine their catalytic activity.

**Figure 4:**
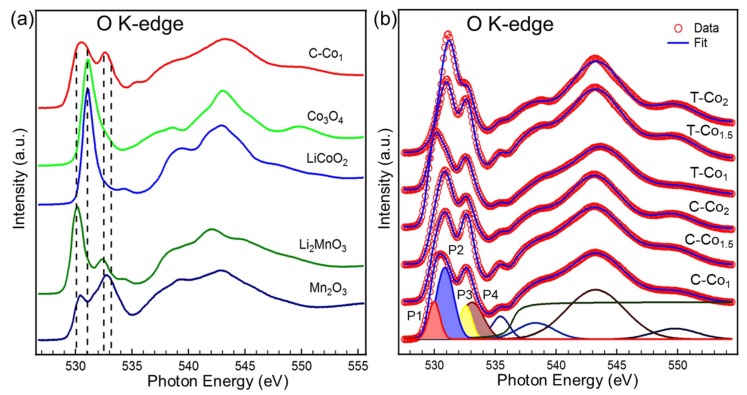
(**a**) O K-edge sXAS spectra of Mn_2_O_3_, Li_2_MnO_3_, LiCoO_2_, Co_3_O_4_, and C-Co_1_. (**b**) O K-edge XAS signals of the spinel oxides; the peak decomposition has also been indicated in the figure. The experimental data are shown with the open red circles and the fitted results are shown with the solid blue line. Shaded Gaussian peaks (P1, P2, P3, and P4) represent the M 3d-O 2p covalency.

The complexity of dual-metal spinels and the abundant features in the pre-edge of O K-edge sXAS spectra inspire us to perform a more in-depth analysis. First of all, we compare a series of reference samples (Co_3_O_4_, LiCoO_2_, Li_2_MnO_3_, Mn_2_O_3_) along with our sample C–Co_1_, as shown in the Figure 4a, to figure out the contributions of the Co^2+^ at tetrahedral site as well as the Co^3+^, Mn^3+^, and Mn^4+^ at octahedral sites to O K-edge spectra. The pre-edge features of Co_3_O_4_ are very similar to the LiCoO_2_, except for an additional weak shoulder at ~532.6 eV corresponding to the Co^2+^ at tetrahedral site. This declares that the contribution of Co^2+^ in the tetrahedron to the O–K pre-edge is negligible in spinel oxides. For Mn-containing oxides, two well-resolved peaks are observed in Li_2_MnO_3_ and Mn_2_O_3_ and their lineshapes are very different. The high-energy peak of Mn_2_O_3_ is broadening and probably the superposition of two peaks [36]. A glancing comparison between the C–Co_1_ and these reference samples illustrates, as shown by the dotted line in the Figure 4a, that the pre-edge of Co_x_Mn_3-x_O_4_ contains all specific features, which can be considered as the spectral fingerprint of Co^2+^ at tetrahedral site as well as the Co^3+^, Mn^3+^, and Mn^4+^ at octahedral sites. Furthermore, we perform a quantitative analysis by spectral fitting method, as shown in Figure 4b (and Figure S1 in Supplementary Materials) [42,43]. In detail, symmetrically constrained Gaussian features and an arctangent function background are employed to do curve fitting. The full width at half maximum (FWHM) and energy position of the Gaussian functions and the arctangent background for the spectra peak deconvolution are listed in Tables S1 and S2 in supplementary materials. The most important information gained from this analysis is that the O–K pre-edge of Co_x_Mn_3-x_O_4_ can be deconvoluted by four well-resolved intense Gaussian features labeled as P1–P4, representing four partial covalency of different Co–O and Mn–O. In addition, their intensities can be determined by the area of the corresponding curve-fitting functions.

**Figure 5:**
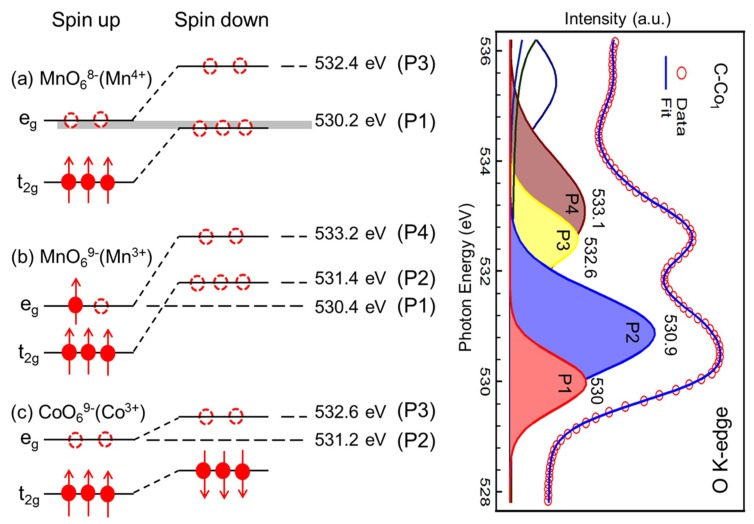
The correlation between four Gaussian peaks of pre-edge portion and partial covalency of specific 3d orbitals. (**Left**) Experimental molecular orbital diagrams based on the XAS spectra. The three clusters, (**a**) MnO_6_^8−^, (**b**) MnO_6_^9−^, and (**c**) CoO_6_^9−^ correspond to Mn^4+^, Mn^3+^, and Co^3+^ in octahedral structure, respectively. (**Right**) O–K pre-edge XAS signals with peak decomposition of C–Co_1_.

To further visualize the correlations between the P1–P4 features and the specific 3d orbitals of Mn^3+^, Mn^4+^, and Co^3+^ in octahedral sites, the quantitative molecular orbital diagram is generated in Figure 5 by considering crystal field and spin states. For high-spin Mn^4+^ (3d^3^, t_2g_^3^) in Li_2_MnO_3_, only two peaks were observed from the transitions to t_2g_-down/e_g_-up (~530.2 eV) and e_g_-down (~532.4 eV) states. For high-spin Mn^3+^ (3d^4^, t_2g_^3^e_g_^1^) in Mn_2_O_3_, triple-peak structure was observed from the transitions to e_g_-up (~530.4 eV), t_2g_-down (~531.4 eV), and e_g_-down (533.2 eV) states [36,40]. For low-spin Co^3+^ (3d^6^, t_2g_^6^) in LiCoO_2_, two-peak structure was observed from the transitions to e_g_-up (~531.2 eV) and e_g_-down (532.6 eV) states. Considering the energy position of the Gaussian peaks and the specific 3d orbital of the references in Figure 5, we assign the partial covalency of specific orbital to the four Gaussian peaks of pre-edge portion. For P2 feature, two sources of partial covalency are Co^3+^ 3d e_g_-up and Mn^3+^ 3d t_2g_-down orbitals. However, the Mn^3+^ 3d t_2g_-down orbital contributes little to this energy position from the O K-edge in Figure 4a. Therefore, the main source of partial covalency is Co^3+^ 3d e_g_-up orbital for P2. Similarly, since Co^3+^ 3d e_g_-down orbital contributes little to the P3 energy position, the main source of partial covalency is the Mn^4+^ 3d e_g_-down orbital for P3. Finally, the relationship between the four Gaussian features and main partial covalency is concluded in Table 1.

**Figure 6:**
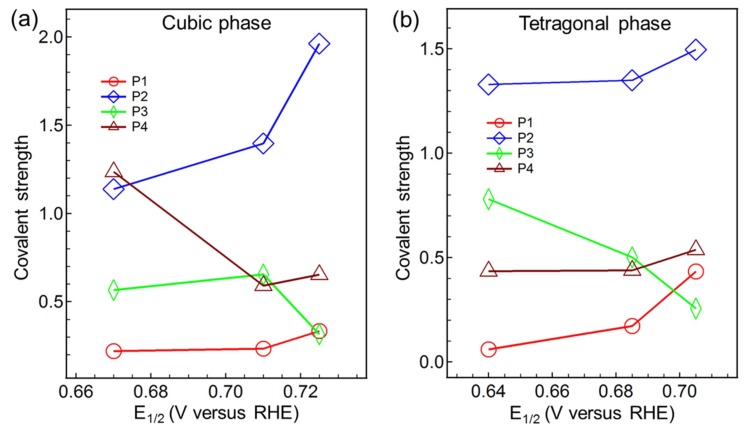
The relationship between the partial M 3d-O 2p covalency strength and ORR catalytic activities. The covalency strength of P1, P2, P3, and P4 versus the half-wave potential E_1/2_ in the cubic (**a**) and tetragonal (**b**) phase.

After distinguishing the partial covalency, its relationship with the ORR activity is analyzed. Since the valence of the metals has little change, the area of the Gaussian feature is mainly determined by the metal ratio and the covalent strength. We normalize the Gaussian feature area to the metal ratio to represent the covalent strength. The relationship between the strength of partial covalency with the half-wave potential E_1/2_ for the cubic and tetragonal spinel oxides shows in Figure 6a,b, respectively. For both cubic and tetragonal phase, increasing the covalent strength of P1 and P2 positively affects ORR activity, while increasing the covalent strength of P3 and P4 negatively affects ORR activity in tetragonal and cubic phase, respectively. This illustrates that the partial covalency of P1 and P2 can boost the ORR catalytic activities, while the partial covalency of P3 and P4 may demote the ORR catalytic activities. As displayed in Table 1, the main origin of the partial covalency of P1 is Mn^4+^ 3d t_2g_-down/e_g_-up and Mn^3+^ 3d e_g_-up orbitals, and P2 is Co^3+^ 3d e_g_-up orbital. That is to say, the enhanced ORR catalytic activities can be attributed to the raised partial covalent strength of O 2p orbital with Mn^4+^ 3d t_2g_-down/e_g_-up, Mn^3+^ 3d e_g_-up, and Co^3+^ 3d e_g_-up orbitals in this spinel system.

Previous researches have demonstrated that the stronger covalent strength of M 3d-O 2p, the more ORR electrocatalytic activity of the oxide can be promoted [19,24], where the covalent strength of M–O are thought to have a critical impact on the rate of desorption and adsorption steps. Our study indicates that not all the covalency of metal 3d orbital and O 2p orbital have a positive push effect on ORR activity. Specifically, the partial covalency of O 2p orbital with Mn^4+^ 3d t_2g_-down/e_g_-up, Mn^3+^ 3d e_g_-up, and Co^3+^ 3d e_g_-up orbitals is benefit for ORR activity. It is consistent with the common sense that the e_g_ orbitals of the metal, rather than the t_2g_ orbitals, interact more easily with the oxygen orbital to produce an adsorbed intermediate with end-on absorption mode [44,45]. In addition, the single electron in Mn^3+^ 3d e_g_-up orbital is also important for the improving catalytic activity. As discussed in previous reports, the existence of a single e_g_ electron in the TM ion is able to form a covalent interaction with the adsorbate [46] and the proper number of e_g_ orbital electron filling can well modulate the balance of intermediates displacement in rate-limiting reactions during traditional four-electron ORR proceeds (Figure S2 in Supplementary Materials) [19,20,21,22]. On account of the above discussion, variations in the ORR performance between Co_x_Mn_3-x_O_4_ (x = 1, 1.5, 2) can be modified by the partial covalency of TM 3d−O 2p by tuning the interaction between spinel catalyst and the molecular oxygen. This may open up a new way to find high-efficiency ORR catalysts by introducing other metals to tune the partial covalent states of M 3d-O 2p.

## 4. Conclusions

In summary, we have studied a substitution strategy to tune the electronic structure of Co_x_Mn_3-x_O_4_ in terms of TM 3d-O 2p covalency, especially the partial covalency between Mn^4+^ 3d t_2g_-down/e_g_-up, Mn^3+^ 3d e_g_-up, and Co^3+^ 3d e_g_-up orbitals with O 2p orbital, for enhancing the ORR activity. The ORR performance increases with the increase of Mn content in both cubic and tetragonal phases. The electronic structure of metal (cobalt and manganese) has very little changes for the substitution of Co by Mn, however, the electronic structure of O can be significantly regulated by studying sXAS. In other words, the factor determining the ORR activity are related to the M 3d-O 2p covalency. More important, the partial covalency between Mn^4+^ 3d t_2g_-down/e_g_-up, Mn^3+^ 3d e_g_-up, and Co^3+^ 3d e_g_-up orbitals with O 2p orbital plays an significant role on the ORR catalytic activities by further analysis of O K-edge, such as integration and peak deconvolution. Our findings demonstrate the key role of partial covalency between specific metal 3d orbitals and O 2p orbital in enhancing the ORR activity, which can provide a new avenue to designing high efficiency catalyst materials for clean energy conversion and storage devices.

## Figures and Tables

**Table 1:** The correspondence between the Gaussian peak and partial covalency.

Gaussian Peak (Energy Position)	Main Partial Covalency
P1 (~530.0 eV)	Mn^4+^ 3d t_2g_-down and e_g_-up orbital, Mn^3+^ 3d e_g_-up orbital
P2 (~530.9 eV)	Co^3+^ 3d e_g_-up orbital
P3 (~532.6 eV)	Mn^4+^ 3d e_g_-down orbital
P4 (~533.1e V)	Mn^3+^ 3d e_g_-down orbital

## Supplementary Materials

The following are available online at https://www.mdpi.com/2079-4991/9/4/577/s1. Figure S1: The O K-edge sXAS signals of the spinel Co_x_Mn_3-x_O_4_ oxides. After subtracting an arctangent background, eight Gaussian functions were employed to fit the sXAS spectrum. Figure S2: Proposed traditional four-electron ORR mechanism on spinel oxide catalysts. The ORR proceeds via four steps: 1, surface oxygen gas adsorption; 2, surface peroxide formation; 3, surface oxide formation; 4, surface hydroxide regeneration. M is a transition-metal cation in octahedral sites. Table S1: The full width at half maximum (FWHM) of the Gaussian functions and the arctangent background (ATAN function) for the O K-edge sXAS spectra peak deconvolution of the cubic and tetragonal spinel oxides (unit: eV). Table S2: The energy position of the Gaussian functions and ATAN function for the O K-edge sXAS spectra peak deconvolution of the cubic and tetragonal spinel oxides (unit: eV).
