# Supplementary material for: Direct Spectroscopy for Probing the Critical Role of Partial Covalency in Oxygen Reduction Reaction for Cobalt-Manganese Spinel Oxides
Source: Nanomaterials (Basel). 2019 Apr 9;9(4):577. doi: 10.3390/nano9040577 (PMC6523907; doi:10.3390/nano9040577)
Supplement: Supplementary file 1 [file nanomaterials-09-00577-s001.pdf]

Supplementary Materials

Direct Spectroscopy for Probing the Critical Role of Partial Covalency in Oxygen Reduction Reaction for Cobalt-Manganese Spinel Oxides

Xinghui Long [1,2,3], Pengfei Yu [1,2], Nian Zhang [1,2], Chun Li [4], Xuefei Feng [5], Guoxi Ren [1,2,3], Shun Zheng [1,2,3], Jiamin Fu [1,2,6], Fangyi Cheng [4] and Xiaosong Liu [1,2,6,*]

[1] State Key Laboratory of Functional Materials for Informatics, Shanghai Institute of Microsystem and Information Technology, Chinese Academy of Sciences, Shanghai 200050, China; xhlong@mail.sim.ac.cn (X.L.); ypfaq@mail.sim.ac.cn (P.Y.); zhangn@mail.sim.ac.cn (N.Z.); gxren@mail.sim.ac.cn (G.R.); shunzheng@mail.sim.ac.cn (S.Z.); fujm@shanghaitech.edu.cn (J.F.)

[2] CAS Center for Excellence in Superconducting Electronics (CENSE), Chinese Academy of Sciences, Shanghai 200050, China

[3] University of Chinese Academy of Sciences, Beijing 100049, China

[4] Key Laboratory of Advanced Energy Materials Chemistry (Ministry of Education) and State Key Laboratory of Elemento-Organic Chemistry, College of Chemistry, Nankai University, Tianjin 300071, China; kemistlic@foxmail.com (C.L.); fycheng@nankai.edu.cn (F.C.)

[5] Advanced Light Source, Lawrence Berkeley National Laboratory, Berkeley, CA 94720, USA; xuefeifeng2013@gmail.com

[6] School of Physical Science and Technology, Shanghai Tech University, Shanghai 200031, China

* Correspondence: xliu3@mail.sim.ac.cn; Tel.: +86-021-6251-1070

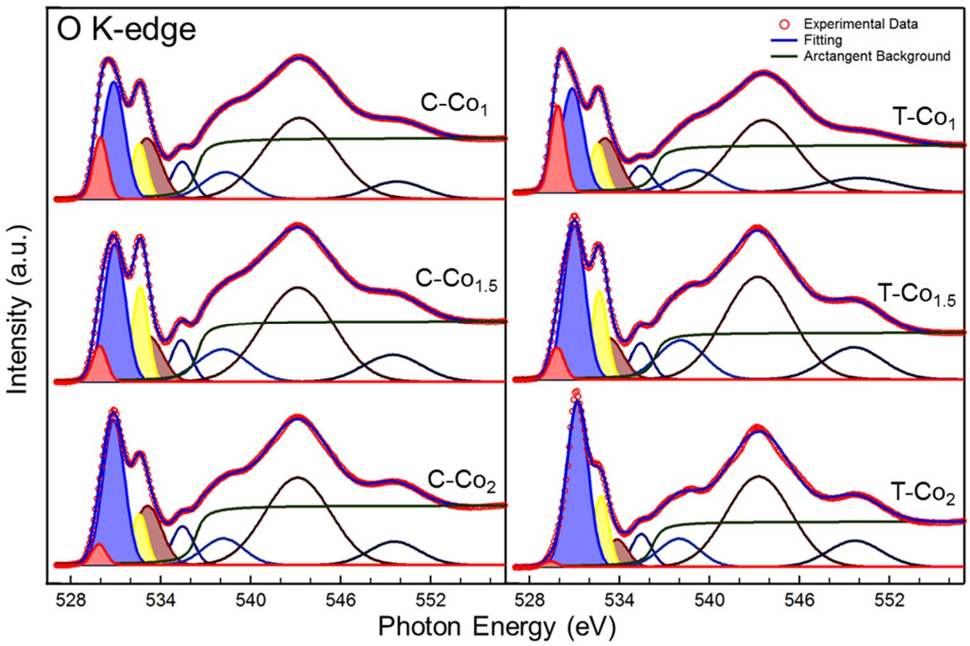

Figure S1. The O K-edge sXAS signals of the spinel $Co_xMn_{3-x}O_4$ oxides. After subtracting an arctangent background, eight Gaussian functions were employed to fit the sXAS spectrum.

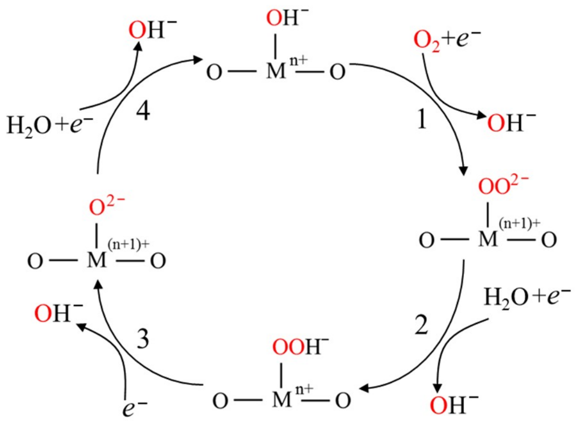

Figure S2. Proposed traditional four-electron ORR mechanism on spinel oxide catalysts. The ORR proceeds via four steps: 1, surface oxygen gas adsorption; 2, surface peroxide formation; 3, surface oxide formation; 4, surface hydroxide regeneration. M is a transition-metal cation in octahedral sites.

Table S1. The full width at half maximum (FWHM) of the Gaussian functions and the arctangent background (ATAN function) for the O K-edge sXAS spectra peak deconvolution of the cubic and tetragonal spinel oxides (unit: eV).

**Sample**	**P1**	**P2**	**P3**	**P4**	**P5**	**P6**	**P7**	**P8**	**ATAN**
C-Co_1	1.118	1.708	1.182	2.226	1.693	3.530	5.633	4.337	1.398
C-$Co_{1.5}$	1.118	1.708	1.182	2.226	1.588	3.995	5.598	4.614	1.398
C-Co_2	1.118	1.708	1.182	2.226	1.690	3.126	5.128	3.988	1.398
T-Co_1	1.118	1.708	1.182	2.226	1.692	3.621	5.290	5.843	1.398
T-$Co_{1.5}$	1.118	1.708	1.182	2.226	1.620	3.670	5.337	4.576	1.398
T-Co_2	1.118	1.708	1.182	1.693	1.567	3.194	5.038	4.210	1.398

Table S2. The energy position of the Gaussian functions and ATAN function for the O K-edge sXAS spectra peak deconvolution of the cubic and tetragonal spinel oxides (unit: eV).

**Sample**	**P1**	**P2**	**P3**	**P4**	**P5**	**P6**	**P7**	**P8**	**ATAN**
**C-Co_1**	529.99	530.87	532.58	533.09	535.44	538.33	543.26	549.77	536.43
**C-$Co_{1.5}$**	529.93	530.93	532.65	533.19	535.39	538.14	543.16	549.48	536.48
**C-Co_2**	529.89	530.87	532.58	533.14	535.47	538.18	543.14	549.58	536.36
**T-Co_1**	529.89	530.86	532.55	533.04	535.44	538.99	543.62	549.99	536.42
**T-$Co_{1.5}$**	529.84	531.00	532.66	533.29	535.43	538.12	543.24	549.63	536.41
**T-Co_2**	529.41	531.20	532.78	533.85	535.47	537.99	543.30	549.70	536.25
